# 3D Finite Element Pseudodynamic Analysis of Deficient RC Rectangular Columns Confined with Fiber Reinforced Polymers under Axial Compression

**DOI:** 10.3390/polym12112546

**Published:** 2020-10-30

**Authors:** Theodora D. Fanaradelli, Theodoros C. Rousakis

**Affiliations:** Laboratory of Reinforced Concrete and Seismic Design of Structures, Department of Civil Engineering, Faculty of Engineering, Democritus University of Thrace (D.U.Th.), 67100 Xanthi, Greece; tfanarad@civil.duth.gr

**Keywords:** finite element method, RC concrete columns, FRP, confinement, RHT model

## Abstract

This paper utilizes the advanced potential of pseudodynamic three-dimensional finite-element modeling to study the axial mechanical behavior of square and rectangular reinforced concrete columns, confined with fiber reinforced polymer (FRP) jackets and continuous composite ropes in seismic applications. The rigorous and versatile Riedel-Hiermaier-Thoma (RHT) material model for concrete is suitably calibrated/modified to reproduce the variable behavior of characteristic retrofitted columns with deficient internal steel reinforcement detailing, suffering nonuniform local concrete cracking and crushing or bulging and bar buckling. Similarly, the 3D FRP jacket or rope confinement models may account for damage distribution, local fracture initiation and different interfacial bonding conditions. The satisfactory accuracy of the reproduced experimental stress-strain envelope behavior enables the analytical investigation of several critical design parameters that are difficult to measure reliably during experiments. Additional parametric analyses are conducted to assess the effects of steel quality. The significant variation of the field of developed strains on the FRP jacket at the ultimate and of the developed strains and deformations on steel cages among different columns are thoroughly investigated. This advanced analytical insight may be directly utilized to address missing critical parameters and allow for more reliable FRP retrofit design of seismic resistant reinforced concrete (RC) columns. Further, it allows for arbitrary 3D seismic analysis of columns (loading, unloading, cyclic or loading rate effects or preloading) or addresses predamages.

## 1. Introduction

Advanced fiber reinforced polymer (FRP) materials made of carbon, glass or aramid fibers, and structural fiber ropes, are widely used in several structural retrofit applications worldwide or as an internal reinforcement [1,2,3,4,5,6,7,8,9,10,11,12,13,14,15,16,17,18]. Concrete columns designed according to early code provisions, take no special care of the effect of strong seismic excitations and corresponding ductility demands or detailing of reinforcement. Existing earthquake-prone columns often need to be strengthened in order to meet the modern design codes. One common technique for the strengthening of these elements is the use of externally bonded FRP sheets. To estimate the effectiveness of this strengthening technique, numerous investigations have been carried out concerning columns with or without internal steel reinforcement having circular cross sections (for example [4,17,18,19,20,21,22]). Significant research has also been done for square/rectangular reinforced concrete (RC) or plain concrete columns under monotonic axial loading [1,23,24,25,26,27,28,29,30] among others. Similar work has been carried out for FRP-confined concrete columns under cyclic axial loading for circular specimens [31,32,33,34,35,36,37] and rectangular ones [2,3,28,35,38,39,40,41,42,43,44,45,46,47,48], with or without transverse and longitudinal steel reinforcement. In a previous work by Parvin et al. [49], there was a literature review on FRP-confined concrete columns strengthened to increase the axial, shear and flexural capacities for a variety of reasons such as lack of confinement, eccentric loading, seismic loading and corrosion. Further, structural fiber ropes have been widely investigated as external or internal reinforcement of columns and RC beams [10,11,12,13,14,15,16,47]. Recent studies have focused on the use of composite fiber ropes made of vinylon, polypropylene or basalt as external reinforcement to confine concrete cylinders [11,12,14] and prisms [13,47]. Additionally, carbon fiber-reinforced polymer ropes have been used for retrofitting beams with near surface-mounted (NSM) and embedded through-section (ETS) methods [15] and as internal transverse shear reinforcement [10,16].

All these experimental investigations have led to the proposal of a large amount of semi empirical models trying to predict with accuracy the ultimate stress and strain behavior of concrete columns when subjected to monotonic axial loading [50,51,52,53,54,55,56,57,58] and few expressions have been proposed to predict the cyclic response [59,60,61,62]. Ozbakkaloglu et al. [63] proposed a probabilistic model for the prediction of ultimate stress and strain of FRP confined columns. In addition to these, the behavior of FRP confined concrete in the microstructural level has been investigated through finite-element (FE) analysis focused on the analysis of solid FRP confined concrete cylinders and rectangular specimens as well [64,65,66,67,68]. Concrete was usually modeled as a Drucker-Prager-type material or as the modified plastic-damage model within the theoretical framework of the concrete damaged plasticity model (CDPM) and a lot of studies have been conducted proposing modified models, trying to reliably predict the behavior of these confined columns.

Yu et al. [64] made a critical review and assessment of the performance of the existing Drucker-Prager (D-P) type concrete plasticity models using both experimental observations and numerical results. They proposed a modified D-P type model, which includes these three features: (a) a yield criterion including the third deviatoric stress invariant; (b) a hardening/softening rule, which is dependent on the confining pressure and (c) a flow rule, which is dependent not only on the confining pressure but also on the rate of confinement increment. Following this work, the same researchers [65] presented a modified plastic-damage model for the modeling of confined concrete including the three features by [64]. Teng et al. [66] presented a 3D FE approach for modeling the behavior of the FRP-confined plain and RC concrete cylinders based on Yu et al.’s plastic-damage model [65]. This FE approach used details such as end restraints and discrete transverse steel reinforcement for the modeling. Earlier, Rousakis et al. [67,68] modeled concrete as a Drucker-Prager-type material, which was suitably modified and calibrated including an advanced and yet simple dilation and damage parameter approach capable of describing hardening and softening behavior of concrete under uniform confinement. Similarly, Karabinis et al. [69] applied this material to 3D FE models of concrete cylinders or RC deficient columns retrofitted with FRPs. The agreement of analytical predictions with experimental behavior of plain and steel reinforced columns, externally confined with FRP was satisfactory in most of the above-mentioned studies.

**Figure 1:**
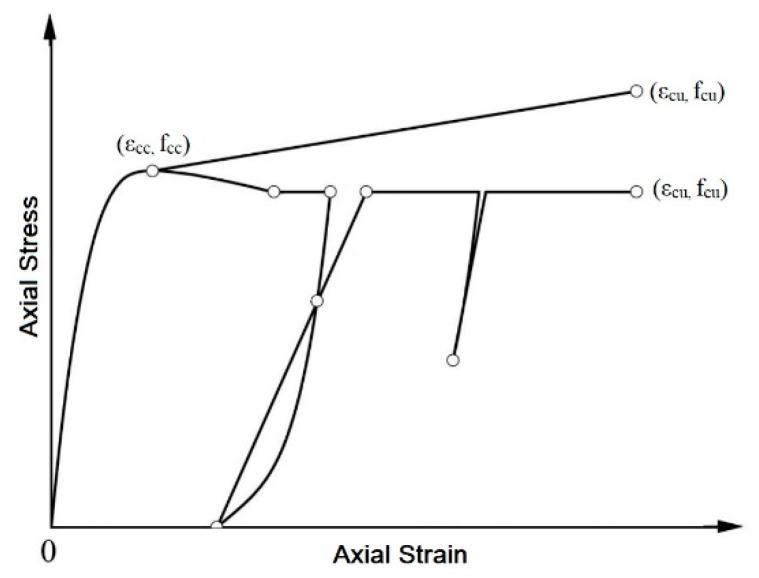
Axial stress-strain curves of unconfined concrete and FRP-confined concretes with ascending and descending second branches.

The extensive investigation of the recent experimental and analytical databases by Fanaradelli et al. [70,71] suggest that several existing design models for plain concrete columns of the rectangular section confined with FRPs could be suitably modified to provide reliably the maximum axial stress and the axial stress at failure for the corresponding concrete columns with internal steel reinforcement. The average absolute error (AAE) of predicted axial stress compared against the experimental results for RC columns under axial concentric compressive monotonic or loading-unloading-reloading cycles of increasing compressive deformation (see Figure 1) was around 15%. In real practice, the seismic redesign of deficient RC columns in existing structures involves highly the prediction of the axial strain at failure (at the ultimate condition). Based on the axial strain ductility of concrete at failure (obtained commonly by axial concentric compression tests on FRP confined plain concrete columns for new structures), the curvature ductility at the section level and the displacement ductility at the member level may be assessed [72,73] among others. However, the AAE of the prediction of the axial strain at failure even for plain concrete non-circular columns confined by FRP under axial compression (monotonic or cyclic) is higher than 50% [70,71]. Further, in existing structures, RC columns may reveal several deficiencies (inadequate internal steel detailing among else) that limit the axial strain at failure. Therefore, it is of high importance to validate the accuracy of any new axial strain models at failure, against the experimental results of RC columns with a variety of geometrical, mechanical and detailing characteristics, aiming at addressing missing critical design parameters.

The approach followed herein is the utilization of several characteristic existing experimental efforts of columns under axial concentric loading-unloading-reloading cycles of increasing compressive displacement (commonly used to assess the axial strain performance of columns in seismic resistant applications [2,3,20,28]) in order to cover the effects of a wide range of critical parameters of the retrofit of RC columns with FRPs (slenderness of bars, sparse stirrups, steel quality and quantity, type and layers of FRP jacket, type of impregnation resins and reinforcing fibers, corner radius, concrete strength, predamages, etc.). Then, these carefully chosen columns are modeled and analyzed for the first time pseudodynamically with three-dimensional finite elements (3D FEs). They provide numerous analytical insights into the effects of different critical parameters to allow for the enrichment of the existing databases with significant missing variables. Special attention is given to take into account the effects of impregnation epoxy polymers used in FRP jackets, with suitable modifications of the properties of the materials. The retrieved characteristic analytical parameters may be directly utilized to propose more reliable design models for the prediction of the failure (ultimate) axial strains of such columns. Therefore, the hybrid experimental-analytical approach is followed (similar to the one already applied successfully in RC beams strengthened with FRPs in shear in Rousakis et al. [74]). Furthermore, the developed versatile and rigorous 3D FE pseudodynamic models may serve for more demanding analyses for seismic resistant applications and preloading effects or to address predamages.

## 2. Materials and Methods

### 2.1. Experimental Database

The final database by Fanaradelli and Rousakis [71] gathers the geometrical and mechanical characteristics of the specimens. It also includes the maximum axial stress of confined concrete at the peak of the stress-strain curve (f_cc_) and the corresponding axial strain (ε_cc_) and the ultimate axial stress (f_cu_) and strain (ε_cu_) of confined concrete at the failure of the column. These characteristic values are depicted in Figure 1. For columns with ascending branches usually there is FRP fracture and thus it was considered that the ultimate stress (at failure) is f_cu_ = f_cc_. For columns with descending second branches, based on the loading control (and loading rate) followed during the tests, very low axial stress values may be recorded at failure. However, based on the existing design recommendations [72,73], the failure of the column was defined at the point (after the maximum bearing axial stress) that the bearing stress drops not lower than 0.85 × f_cc_ (=f_cu_, ultimate axial stress). Therefore, the failure (ultimate) stress may be f_cu_ = 0.85 × f_cc_ if there is a gradual load drop (because of inadequate FRP confinement or longitudinal bar buckling, etc.) or higher than 0.85 × f_cc_ and lower than f_cc_ if there is an abrupt FRP fracture. Based on this approach the corresponding ε_cc_ and ε_cu_ are defined at the same points, see also [17,19,53,58,63].

As already mentioned, the prediction of ε_cu_ for all the columns of the corresponding database presents AAE higher than 50%. This paper suggests that the gathered characteristics and experimental results are not adequate in order to accurately predict the strain at failure. Therefore, 3-dimensional FE models of characteristic columns were developed and analyzed pseudodynamically, aiming at addressing unidentified design parameters through thorough assessment of the analytical results at regions that the corresponding experimental results were not available or impossible to measure (see a similar approach in [74]). RC square and rectangular concrete columns externally confined with FRP materials, subjected to cyclic axial compressive load were selected from the studies by Rousakis and Karabinis [2], Ilki et al. [20] and Isleem et al. [28]. All the test specimens include conventional longitudinal and transverse steel reinforcement. The columns were externally confined with Carbon Fiber Reinforced Polymers (CFRP) jackets and the direction of the fibers was perpendicular to the column axis. The geometrical and the nominal mechanical data for the 11 chosen specimens are presented in Table 1, following the labels of the original papers.

### 2.2. Pseudodynamic Finite Element Models and Analyses

The rigorous and versatile 3-dimensional pseudodynamic finite-element modeling was used for the first time to study the axial mechanical behavior of square and rectangular RC columns, confined with FRP jackets and continuous composite ropes for seismic resistant applications. Advanced material models for concrete, composites and steel are suitably calibrated to conduct numerous analyses. ANSYS Explicit Dynamics [75] was used to construct the advanced models, execute the analyses and for post-processing of results.

#### 2.2.1. Concrete

The Riedel-Hiermaier-Thoma (RHT) model [76,77,78] was used (ANSYS Autodyn [75]). RHT is a constitutive model for brittle materials and is a combined plasticity and shear damage model in which the deviatoric stress in the material is limited by a generalized failure surface of the form:

(1) $$f\left(P,\sigma_{\mathrm{eq}},\theta ,\dot{\varepsilon }\right)=\sigma_{\mathrm{eq}}-Y_{\mathrm{TXC}\left(P\right)}\times F_{\mathrm{CAP}\left(P\right)}\times R_{3\left(\theta \right)}\times {\left(F\right)}_{\mathrm{RATE}\left(\dot{\varepsilon }\right)}$$

This failure surface can be used to represent the following aspects of the response of geological materials: pressure hardening, strain hardening, strain rate hardening in tension and compression, third invariant dependence for compressive and tensile meridians, strain softening (shear induced damage) and coupling of damage due to porous collapse. It enables the reproduction of monotonic, repeated or cyclic imposed displacements (usually met in seismic resistant assessment or retrofit of columns) highly inelastic behaviors, damage redistribution effects and highly local degrading behavior. The RHT model was suitably calibrated through extensive and comprehensive investigations to eliminate the effect of the rate of axial loading and to provide satisfactory hardening or softening or crashing behavior of concrete under triaxial loading and the effects of predamaged concrete core and damage redistribution. Table 2 includes the used values for the compressive concrete strengths.

#### 2.2.2. Longitudinal and Transverse Steel

In these pseudodynamic analyses, plastic deformation is computed by reference to the Von Mises yield criterion. Therefore, the local yield condition is

(2) $${\left(\sigma_{1}-\sigma_{2}\right)}^{2}+{\left(\sigma_{2}-\sigma_{3}\right)}^{2}+{\left(\sigma_{3}-\sigma_{1}\right)}^{2}=2\Upsilon^{2}$$

where Y is the yield stress in simple tension.

The onset of yielding (plastic flow) is purely a function of the deviatoric stresses (distortion). A bilinear isotropic hardening model was used to define the yield stress (Y) as a linear function of plastic strain, ε_p_

(3) $$\Upsilon =\Upsilon_{0}+A\times \varepsilon_{p}$$

where Y_0_ is the yield strength and A the tangent modulus.

Table 2 includes the used values for the yield stress of the steel bars.

#### 2.2.3. Fiber Reinforced Polymer Jacket

The model for structural reinforced polymer composites was suitably calibrated taking into consideration the common properties of epoxy resins used for fiber sheet impregnation and bonding on concrete. Therefore, the analyses may reproduce local damage initiation of the jacket (local fracture) and thus consider the effects of the corner radius of the section. Full bonding of the jacket on concrete was considered in all cases (debonding is not critical for the confinement applications under consideration). As the fibers in the FRP jackets, considered in the present study, were all oriented in the hoop direction, the stiffness (both tensile and compressive) in the axial direction (i.e., the loading direction of the column) was negligibly small. Therefore, the elastic lamina option was used to model an orthotropic elastic material. A local coordinate system was assigned to the FRP jacket, and the hoop direction and the axial direction were adopted as the first principal and second principal material orientations, respectively.

The modulus of elasticity in the axial direction and the shear modulus were both assumed to have lower values than the ones provided by the manufactures for the net fiber sheet, because they take into account the impregnation with the epoxy resin. For example, for the carbon fibers for specimen series BS1C/BS2C of Table 1, the manufacturer’s elastic modulus was 240,000 MPa. The orthotropic elasticity properties used for the analyses were reduced to 59,160 MPa for the modulus of elasticity in the directions perpendicularly to global loading (along the direction of carbon fibers). The thickness of the jacket was suitably increased to 0.475 per layer, taking into account the thickness of the epoxy resin. The modulus of elasticity multiplied by the thickness of the jacket, in both cases (considering the resin or not) gives the same axial rigidity value. Further, the jacket was modeled with the mechanical properties of the resin alone at the direction parallel to global loading. The suitable consideration of the resin besides the reinforcing fibers may reproduce the realistic damage accumulation in the composite jacket. Table 2 presents the used values. Displacement compatibility was considered between concrete and composite material at their interface.

FE analysis includes the following steps: (1) the geometric characteristics of the members of the model were determined, (2) the properties of the materials were defined, (3) the connections and contacts among members were assigned and (4) the type of discretization of the model and the density of discretization was determined depending on the number of seeds in each surface and the suitable meshing technique (always dependent on the specific application). Seeds are constant nodes, placed along the perimeter of the cross sections. They control the precise location of the meshing nodes. Thus, special care was taken for the seed distribution, in order to take into account, the curvature of the edges and crucial detailing of the specimens. The main target of meshing is the compatibility of the nodes between the concrete core and the FRP jacket in order to avoid convergence problems during analysis, (5) the mode of imposed displacement over time was defined to simulate pseudodynamic application. Monotonic axial displacement was forced at the top of the column, directly on the concrete core and not on the FRP jacket and (6) the boundary conditions of the model were determined. “Fixed Support” was chosen at the base of the column. The monotonic mode can provide the envelope analytical load-displacement curve and thus avoid the extremely time-consuming cyclic mode, as the experimental response between identical columns subjected to monotonic or cyclic loading was fairly close to each other.

#### 2.2.4. Boundary Conditions and Contacts

Two body interactions were created. All the elements adopted the “Frictionless” type. This type simulated friction conditions between all the elements. For the second “Body Interaction”, only the elements that made up the longitudinal and transverse reinforcement were selected and for the integration of the reinforcement within the concrete core. When an element consists of several parts, it is necessary to determine the connections between the elements. Based on numerous investigations with five available contact options, the interaction surface between the FRP and the concrete that is in contact was considered fully “bonded” for all specimens. All the nodes of bodies included in bonded interactions were tied to faces of involved bodies.

#### 2.2.5. Mesh

**Figure 2:**
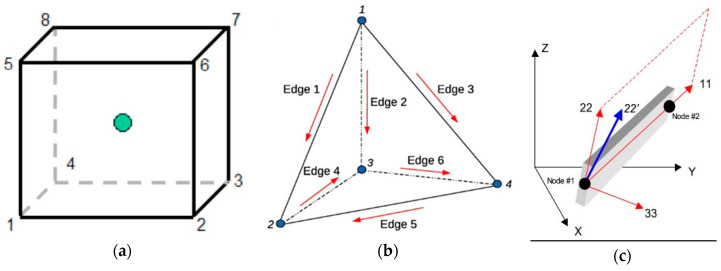
(**a**) “Hexahedral Solid Elements”; (**b**) “Tetrahedral Solid Elements” and (**c**) “Beam (Line) Elements”.

Explicit mesh was followed to form accurate 3D grid. Six-node elements (hexahedral solid elements; Figure 2a were considered for the concrete body. In addition, at the corner regions of the concrete bodies, four-node tetrahedral solid elements were used to efficiently model the curvature of the edges (Figure 2b). The CFRP sheets were modeled similarly with hexahedral and tetrahedral solid elements to account for their global and local effects. For the internal steel reinforcement 2-node beam (line) elements (Figure 2c) were chosen, with the potential to develop large axial deformations.

#### 2.2.6. Element Sizing

**Figure 3:**
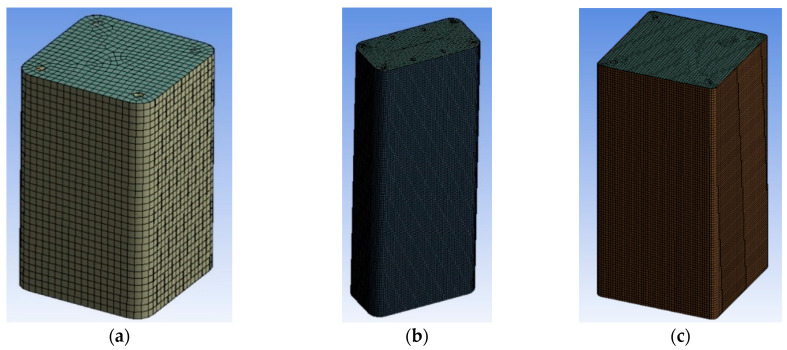
Mesh of specimens: (**a**) Series BS1C/BS2C by [2]; (**b**) Series R2.0H2CL by [28] and (**c**) Series LSR-R-1-3 by [20].

Dense meshing is necessary mostly because of the dimensions of the corner radius and to better introduce the interactions of the steel reinforcement cage and of the FRP jacket with the concrete core. These aspects are very important for FRP retrofitted large scale specimens with internal steel reinforcement in order to better reproduce the observed modes of failure. Therefore, for specimen series BS1C and BS2C by [2], the average element size was 10 mm (Figure 3a). The element size for specimens R2.0H2CL3 and R2.0H2CL4 by [28] was 5 mm (Figure 3b). For specimen LSR-R-1-3-10b by [20], the element size was again 5 mm (Figure 3c). A similar element size was chosen for specimens LSR-R-1-3-20b and LSR-R-1-3-40b by [20]. For all these columns, detailed sensitivity analyses with 15 mm and 25 mm element sizes were also conducted (not presented herein).

## 3. Analytical Results

### 3.1. Stress-Strain Curves

**Figure 4:**
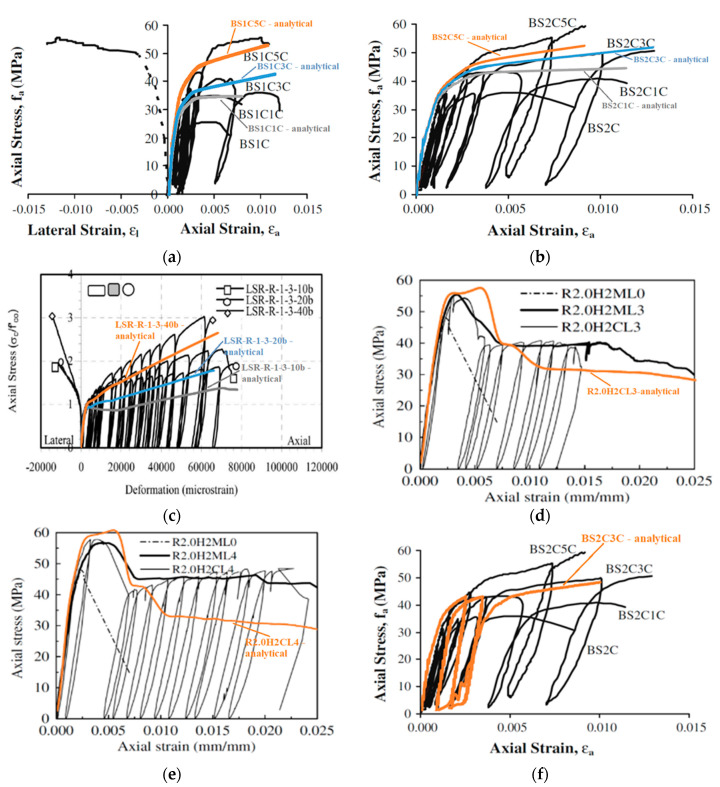
FE analysis predictions versus experimental stress-strain results for (**a**) specimen series BS1C by [2]; (**b**) specimen series BS2C by [2]; (**c**) specimen series LSR-R-1-3 by [20]; (**d**) specimen R2.0H2CL3 by [28]; (**e**) specimen R2.0H2CL4 by [28] and (**f**) specimen BS2CL3 by [2].

The monotonic numerical analyses can serve as the envelope stress-strain curves of the experimental ones (imposed to repeated loading-unloading of increasing imposed displacement) and present satisfactory convergence with the experimental results up to the ultimate conditions of the column series BS1C, as shown in Figure 4a. The analysis for specimen BS1C1C provides the ultimate stress of 34.99 MPa compared to the experimental one of 31.74 MPa at the experimental axial strain of 0.00795. Specimen BS1C3C presents an ultimate axial stress of 42.65 MPa at a strain of 0.01163, while the experimental stress was 34.85 MPa. The numerical analysis presents a good convergence with the experimental results for ultimate conditions of the specimen BS1C5C providing 53.10 MPa, compared with a corresponding experimental stress of 53.60 MPa at a strain of 0.01083.

The performance of the FE analytical models for the columns with steel stirrups spaced at 95 mm (BS2C series) was similar, as presented in Figure 4b. Specimen BS2C1C confined with 1 layer of CFRP shows the ultimate analytical stress of 44.81 MPa, while the experimental stress was 39.37 MPa at the strain of 0.01144. For specimen BS2C3C confined with 3 layers of CFRP the ultimate stress from the FE analysis was 52.18 MPa at a strain of 0.0129 whilst the experimental ultimate stress was 50.68 MPa. Finally, the FE analysis for specimen BS2C5C confined with 5 layers of CFRP presents a good convergence with the experimental ultimate conditions, providing the analytical stress of 52.76 MPa compared to the experimental one of 59.21 MPa, at a strain of 0.00917.

The performance of the FE models against the experimental behavior of RC columns of the square concrete section of very low concrete strength and with a different corner radius with steel reinforcements of intermediate quality are presented in Figure 4c. For specimen LSR-R-1-3-10b confined with 3 layers of CFRP, at the ultimate condition, the experimental stress was 26.6 MPa and the analytical was 21.31 MPa, at a strain of 0.078. For specimen LSR-R-1-3-20b the ultimate experimental stress was 31.21 MPa and the analytical was 28.35 MPa, at a strain of 0.066. Finally, for specimen LSR-R-1-3-40b the experimental stress was 48.07 MPa and the analytical was 42.27 MPa, at the ultimate strain of 0.068.

The comparisons for the RC columns of rectangular section with steel reinforcements of lower quality and sparse stirrups are presented in Figure 4d,e. For specimen R2.0H2CL3 confined with 3 layers of CFRP, at the ultimate condition, the experimental stress was 46.24 MPa and the analytical was 58.85 MPa at a strain of 0.005. For specimen R2.0H2CL4 confined with 4 layers of CFRP, at the ultimate condition, the experimental stress was 49.13 MPa and the analytical was 58.76 MPa at a strain of 0.006. Figure 2 suggests the advanced FE models can capture the general stress-strain behavior with hardening or softening post-elastic branches, recorded in the experiments. The accuracy of the prediction of the stress levels at different strain levels and at the ultimate condition conditions was also satisfactory. Figure 4f also suggests that the FE models could capture with great accuracy the loading and unloading path of these cyclic axial loaded columns.

### 3.2. Characteristic Concrete Damage and FRP and Steel Deformation Variation

Based on the advanced and reliable FE analyses, several aspects of the behavior of the retrofitted RC columns may be further investigated. The RHT model for concrete may provide quantification of the local damage initiation, extent and development within the concrete core through the index named after the damage coefficient (0 for no damage and 1 for full damage or for 100% damage, i.e., tensile or compressive, etc., in the 3-dimensional stress-strain state) at different levels of imposed displacements. Figure 3 presents the variation of the RHT concrete damage coefficient at the whole concrete body and at characteristic section levels (at and in between the steel stirrups). Further, the tensile strains on the FRP jacket were evaluated at the same characteristic levels and at the corner of the sections or at the middle of the sides. These characteristic areas may capture the significant variation of the FRP strains all over its interface with the concrete. This variable tensile strain field on the FRP is owed to the deformed internal steel cage and to the non-circular concrete section. Finally, the FE analyses provided the corresponding variation of strains on the steel bars and stirrups, and their lateral deformations at characteristic positions.

#### 3.2.1. Specimen Series BS1C

**Figure 5:**
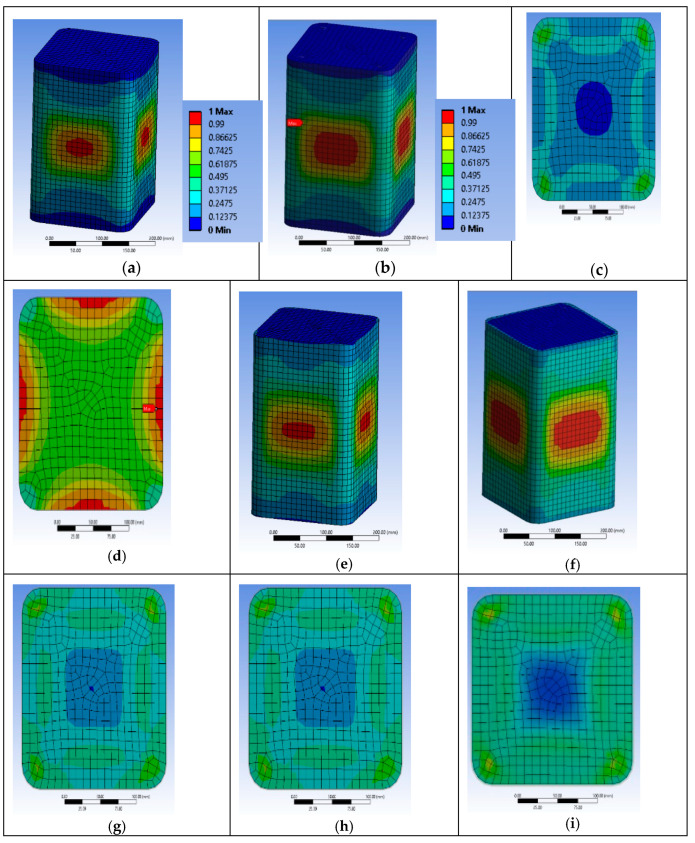
Variation of the concrete damage of specimen BS1C1C (**a**) one step after the first concrete damage; (**b**) at the ultimate condition; (**c**) at the level of the stirrup and (**d**) between two stirrups; BS1C3C (**e**) one step after the first concrete damage; (**f**) at the ultimate condition; (**g**) at the level of the stirrup and (**h**) between two stirrups and BS1C5C (**i**) at the ultimate condition.

**Figure 6:**
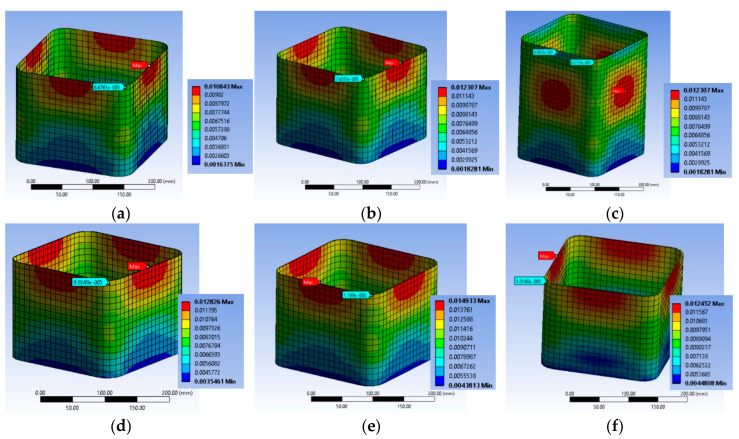
Variation of the Carbon Fiber Reinforced Polymers (CFRP) strain and characteristic values of specimen BS1C1C (**a**) one step after the first concrete damage at the mid-height of the column; (**b**) at the ultimate condition at the mid-height of the column and (**c**) at the ultimate condition at the level of the stirrup; BS1C3C (**d**) one step after the first concrete damage at the mid-height of the column and (**e**) at the ultimate condition at the mid-height of the column and (**f**) BS1C5C at the ultimate condition at the mid-height of the column.

The FE analyses suggest that the first local damage for BS1C1C initiated at the concrete located at the middle-side regions of the specimen at its middle-height, between the two steel stirrups and the FRP jacket (Figure 5a) at 0.00703 axial strain. The strain of the FRP at the mid-height of the specimen was about 0.0108 (less than the material failure; Figure 6a). For the ultimate total strain of the specimen of 0.00795, significant damage spreading into the concrete was observed in its middle area. Figure 5b–d shows the critical area where the highest damage coefficient occurred. At the final step, the maximum strain of the FRP was about 0.0123, which was also less than its capacity (Figure 6b,c).

Similarly, specimen BS1C3C shows first local damage in the concrete at the center of the specimen, between the two steel stirrups and the FRP jacket (Figure 5e) at the 0.009375 axial strain of concrete. The highest tensile strain of the FRP was located in the same area (Figure 6d) around 0.0128, lower than its capacity. Figure 5f–h shows the critical area where the highest degree of damage occurred at failure. In this final step, at the 0.01163 axial strain of concrete, the corresponding tensile strain of the FRP was about 0.0149, very close to its capacity (Figure 6e). Specimen BS1C5C confined with 5 layers of CFRP behaved differently than the other two columns of this series. During the last step of the axial loading, there was extremely high damage coefficient at the mid-height of the specimen, but the concrete, despite being close to, did not exceed the total local damage initiation (Figure 5i). The highest CFRP strain for all reference points was 0.0125, less than the material strain limit of 0.0155 (Figure 6f).

These analyses suggest that in experiments the effects of slender bars could be more pronounced (for the FRP jacket to reach its capacity) as the bars are in reality self-anchored inside the column and not fixed on the bottom and top boundaries as in the FE models. However, no unstable behavior of the bars under compression was recorded in the FE analyses up to the ultimate axial strain. It seems that there might be an interaction between the local damage of the concrete and the FRP fracture that needs further experimental and numerical investigation.

**Figure 7:**
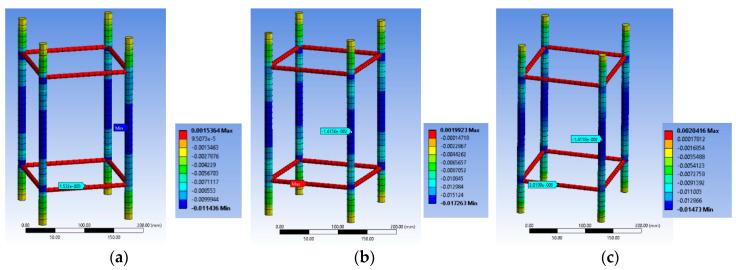
Variation of ε_stirrup_max_, ε_long_mid_ and ε_long_min_ for specimen (**a**) BS1C1C; (**b**) BS1C3C and (**c**) BS1C5C.

Figure 7a–c highlights the corresponding deformational state of the steel cage for the BS1C series. For specimen BS1C1C the maximum stirrup strain was 0.001532, for BS1C3C it was 0.001992 and for BS1C5C it was 0.00202. It seems that the maximum compressive strain on the slender bars was higher than the concrete average axial strain at the ultimate condition for all three different levels of confinement and the stirrup strain was close to yielding. Additionally, the minimum strain of the longitudinal steel bars and the strain at their middle point coincided for all specimens. So, for column BS1C1C this strain was −0.01136, for specimen BS1C3C it was −0.016156 and for BS1C5C it was −0.014118.

#### 3.2.2. Specimen Series BS2C

**Figure 8:**
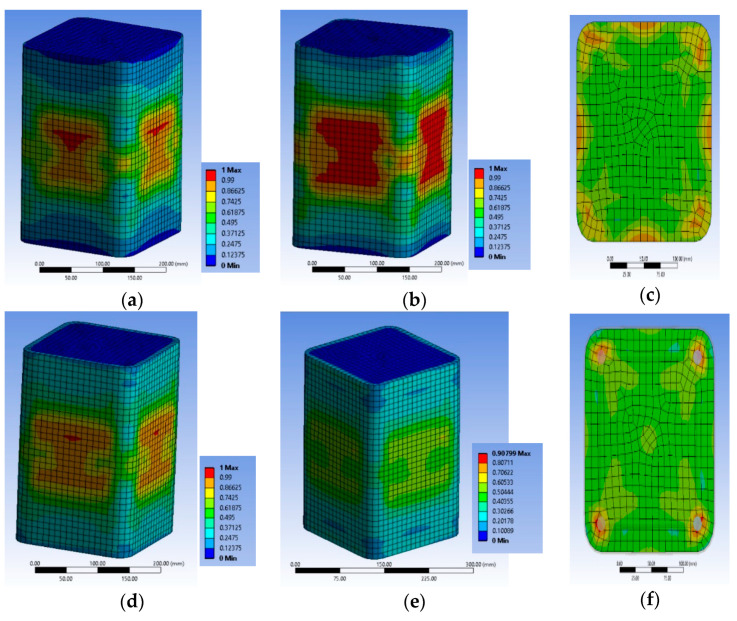
Variation of concrete damage of specimen BS2C1C (**a**) one step after the first concrete damage; (**b**) at the ultimate condition and (**c**) at the level of the stirrup; BS2C3C (**d**) at the ultimate condition and BS2C5C (**e**) at the ultimate condition and (**f**) at the level of the stirrup.

**Figure 9:**
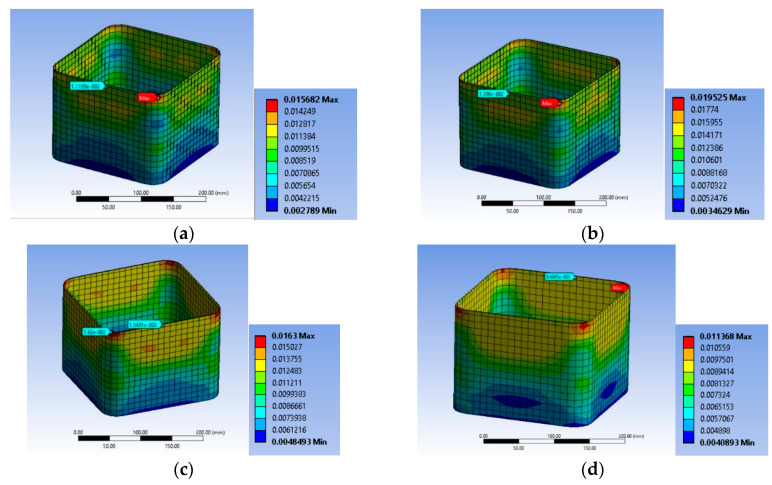
Variation of the CFRP strain and characteristic values of specimen BS2C1C (**a**) one step after the first concrete damage at the level of the stirrup and (**b**) at the ultimate condition at the level of the stirrup; BS2C3C (**c**) at the ultimate condition at the level of the stirrup and (**d**) BS2C5C at the ultimate condition at the level of the stirrup.

The analytical elaboration for the specimens BS2C1C and BS2C3C with stirrups at 95 mm spacing (BS2C series) shows that the concrete failed locally and the FRP also reached its local failure. For BS2C1C, for axial strain of 0.009375, the concrete local failure started at the corner region of the mid-height stirrup (Figure 8a–c). At this point the strain of the FRP was about 0.0156 (Figure 9a). When the total strain of the specimen was 0.01144, significant damage in the concrete was observed in its middle-height area. The damage accumulated around the mid-height stirrup and extended in the area above and below it. Figure 8b shows the critical area where the highest degree of damage occurred (more than 90% of full damage). At the final step, the strain of the FRP was higher than the material failure in the FE analyses (Figure 9b).

The FE results for BS2C3C were similar with the initiation of local failure within the concrete but without simultaneous local FRP failure. However, at the ultimate condition, the damage spread further within the concrete and FRP strain at the ultimate condition was again higher than its capacity (Figure 8d and Figure 9a–c).

Specimen BS2C5C behaved differently than the other two specimens of this series. Specimen BS2C5C had 5 layers of CFRP and the concrete did not exhibit local damage. It is observed that in the last step of the loading, there was severe damage at the corners of the middle stirrup, but the concrete did not fail locally (Figure 8e,f). The CFRP’s strain for all reference points was less than 0.0155 (Figure 9d). It is observed that the specimen confined with the 5 layers of CFRP (BS2C5C) did not suffer any severe crushing in the concrete core. On the contrary, BS2C1C was crushed to a great extent over its perimeter. It is also observed that the highest strain of the CFRP was developed at the corner regions, at the middle stirrup on all three specimens.

The ratio of strain at the corner of the middle stirrup to strain of the corner in-between the two stirrups in the BS2C1C specimen was close to 3, while in the other two specimens the same ratio was about 2. This is a consequence of the first development of concrete cracks at the corner, at the level of the middle stirrup as is shown in the corresponding figures. The ratio of the maximum strain at the corner, at the level of the middle stirrup to the minimum strain of the mid-side at the same level for all these specimens, was close to 1. It seems there was more uniform strain distribution along this axis for the three specimens. It seems that there was a more pronounced interaction between the local damage of the concrete and the FRP fracture at the corners that needs further experimental and numerical investigation.

**Figure 10:**
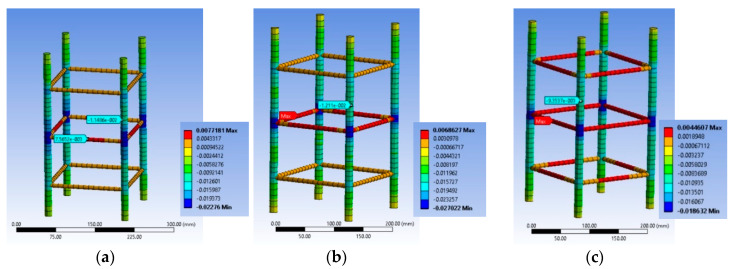
Variation of ε_stirrup_max_, ε_long_mid_ and ε_long_min_ for specimen (**a**) BS2C1C; (**b**) BS2C3C and (**c**) BS2C5C.

Figure 10a–c highlights the corresponding deformational state of the steel cage for BS2C series. It seems that the maximum compressive strain on the slender bars was far higher (around 2 times) than the concrete average axial strain at the ultimate condition for all three different levels of confinement and located at the stirrup level. In particular, for specimen BS2C1C this strain was −0.02276, for BS2C3C it was −0.027022 and for BS2C5C it was −0.01823. Thus, the axial strains of the bars at the level in-between the stirrups were close to the average axial strains of concrete. In particular, for specimen BS2C1C this was is −0.011486, for BS2C3C it was −0.01211 and for BS2C5C it was −0.009354. The stirrup strains were far beyond the yielding in all cases. For specimen BS2C1C it was 0.007565, for BS2C3C1 it was 0.006863 and for BS2C5C it was 0.00461.

#### 3.2.3. Specimen Series LSR-R-1-3

**Figure 11:**
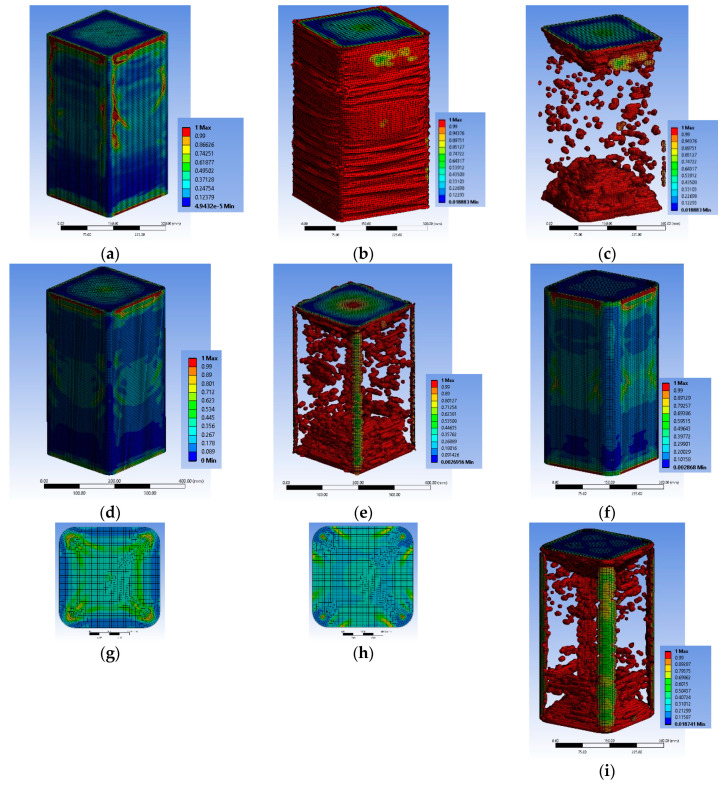
Variation of concrete damage of specimen LSR-R-1-3-10b (**a**) one step after the first concrete damage; (**b**) at the ultimate condition and (**c**) without areas with damage; LSR-R-1-3-20b (**d**) one step after the first concrete damage and (**e**) without areas with damage and LSR-R-1-3-40b (**f**) at the ultimate condition; (**g**) at the level of the stirrup; (**h**) between two stirrups and (**i**) without areas with damage.

**Figure 12:**
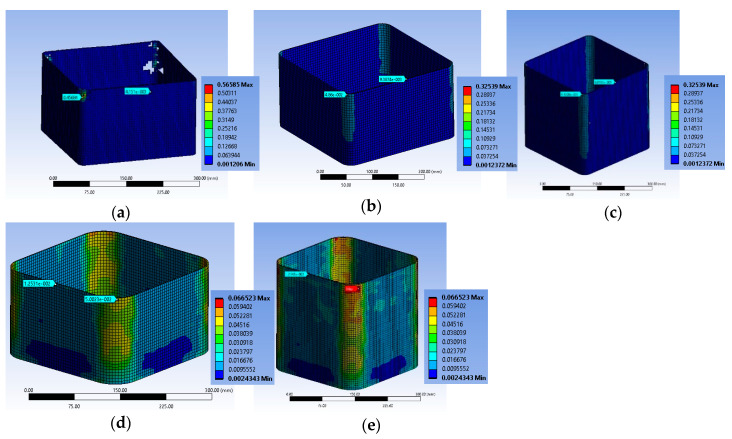
Variation of the CFRP strain and characteristic values of specimen LSR-R-1-3-10b (**a**) at the ultimate condition at the level of the stirrup; LSR-R-1-3-20b at the ultimate condition (**b**) at the level of the stirrup and (**c**) at the mid height of the specimen and LSR-R-1-3-40b at the ultimate condition (**d**) at the level of the stirrup and (**e**) at the mid height of the specimen.

The FE analyses for specimen LSR-R-1-3-10b, with a very low corner radius of 10 mm, suggest that the first local damage initiates at the concrete located at the four corners of the specimen at the level of the stirrup and it is extended to more than the half height of it. The first local damage was shown for an axial strain of 0.045 (Figure 11a). It is observed that the concrete was completely damaged during the axial loading and up to the ultimate axial strain of 0.07767 (Figure 11b,c). There was a more or less uniform distribution of the CFRP strain along the height of the specimen LSR-R-1-3-10b. However, there were multiple regions where the FRP jacket was locally bulged and fractured, because of the local concrete damage (multiple bulging of concrete) and the tensile strains by far exceeded its capacity (Figure 12a).

The FE analytical behavior of column LSR-R-1-3-20b was similar. The concrete local failure initiated at the 0.033 axial strain and it was located at the same concrete area (Figure 11d). The concrete was completely damaged during the axial loading and up to the ultimate axial strain of 0.066 (Figure 11e). There were multiple regions that the FRP jacket was locally bulged because of the local concrete damage (multiple bulging of concrete) and the tensile strains by far exceeded their capacity (Figure 9b,c).

Specimen LSR-R-1-3-40b shows similar damage development inside the concrete core (Figure 11f–h). There were again multiple regions that the FRP jacket was locally bulged because of the local concrete damage (multiple bulging of concrete) but outside of the corner region (Figure 11i and Figure 12d). Again, the tensile strains by far exceeded its capacity at the corner region but in this case of a high corner radius the tensile strain was higher at the corner area at the level in-between the stirrups (Figure 12d,e). In this series, the interaction between the local damage of the concrete and the FRP fracture was more pronounced. This may be attributed to the low concrete strength and low corner radius of the concrete section.

**Figure 13:**
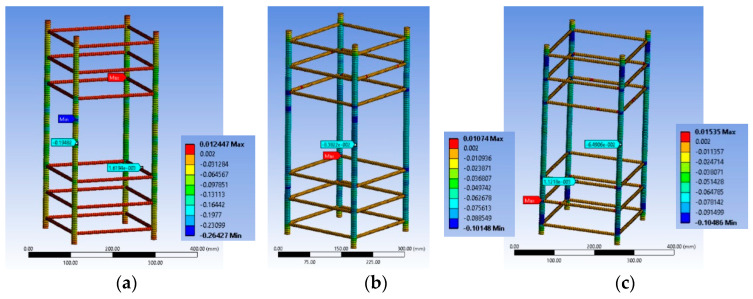
Variation of ε_stirrup_max_, ε_long_mid_ and ε_long_min_ for specimen (**a**) LSR-R-1-3-10b; (**b**) LSR-R-1-3-20b and (**c**) LSR-R-1-3-40b.

Figure 13a–c highlights the corresponding deformational state of the steel cage. It seems that the maximum compressive strain on the slender bars was extremely high for the specimen with 10 mm corner radius and denotes unstable behavior of the bars due to the severely damaged concrete and FRP at the corner region. The axial strains of the bars at the level in-between the stirrups were far higher than the average axial strains of concrete for 10 mm corner radius (−0.19194). However, the same axial strains for the bars in specimens with a 20 mm (−0.08103) and 40 mm (−0.06685) corner radius were closer to the average axial strains of the columns. The stirrup strains were similar in all cases and higher than the yielding ones in all cases (for LSR-R-1-3-10b it was 0.01245, for LSR-R-1-3-20b it was 0.01074 and for LSR-R-1-3-40b it was 0.01535).

#### 3.2.4. Specimen Series R2.0H2CL

**Figure 14:**
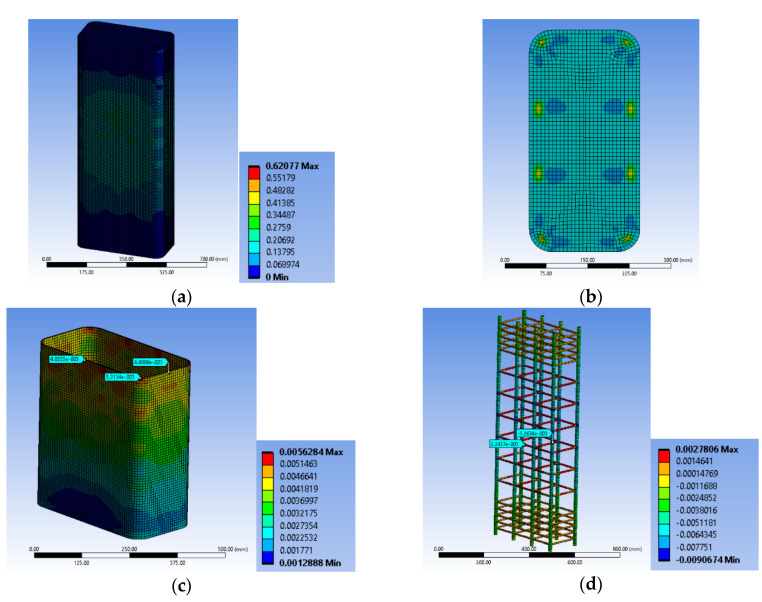
Variation of concrete damage (**a**) at the ultimate condition condition; (**b**) at the level of the stirrup; (**c**) CFRP strain and characteristic values at the ultimate condition at the level of the stirrup and (**d**) ε_stirrup_max_, ε_long_mid_ and ε_long_min_ for specimen R2.0H2CL3.

The FE analysis shows that local damage initiated at the concrete located at the area around the longitudinal steel bars at the level of the stirrup (Figure 14a,b) in specimen R2.0H2CL3. The tensile FRP strain was higher at the corners of the specimen at the level of the stirrup, as in specimens of the BS2C series (Figure 14c). This analysis had a stress-strain curve with a descending inelastic (second) branch (i.e., f_cu_ < 0.85 × f_cc_ < f_cc_) and the ultimate stress f_cu_ was less than 15% of the maximum bearing stress. So, there was a revision of the ultimate value to the 0.85 × f_cc_ and of the corresponding value for ε_cu_ compared to the experimental stress-strain curves of the test results. The analytical results were evaluated for the ultimate axial strain equal to 0.005.

**Figure 15:**
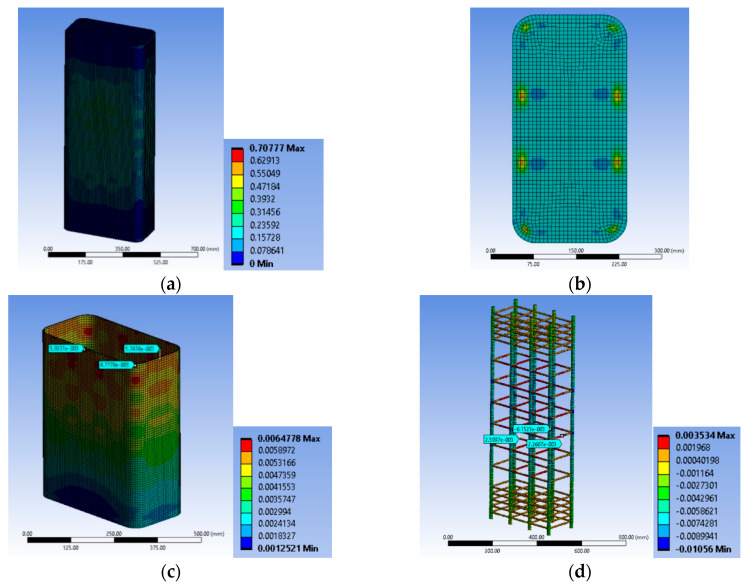
Variation of concrete damage for specimen (**a**) at the ultimate condition; (**b**) at the level of the stirrup; (**c**) CFRP strain and characteristic values at the ultimate condition at the level of the stirrup and (**d**) ε_stirrup_max_, ε_long_mid_ and ε_long_min_ for specimen R2.0H2CL4.

Specimen R2.0H2CL4 exhibited similar behavior to the previous column. The concrete area located around the longitudinal steel bars at the level of the stirrup was crucial (Figure 15a,b). The highest FRP strain was recorded at the corners of the specimen at the level of the stirrup (Figure 15c). The analytical results were similarly evaluated for an axial strain equal to 0.006.

It seems that in both cases, due to the high side ratio (equal to 2), the effectiveness of the confinement provided by the steel stirrups and the FRP jacket was too low to enhance the deformability and strength of the plain concrete or to restrict the unstable behavior of slender steel bars. Therefore, there was a degrading post-peak stress-strain behavior and the bearing stress was more or less stabilized at very low levels for structural applications (lower than (0.80–0.85) × f_cc_). The maximum tensile strains on the FRP were similarly low around 0.004–0.0064.

Figure 14d and Figure 15d highlight the corresponding deformational state of the steel cage. It seems that the maximum compressive strain on the slender bars was far higher (around 2 times) than the concrete average axial strain at the ultimate condition for the two different levels of confinement and located at the stirrup level. The axial strains of the bars at the level in-between the stirrups were close to the average axial strains of concrete (for R2.0H2CL3 it was −0.005263 and for R2.0H2CL4 it was −0.006152). The stirrup strains were far beyond the yielding in all cases (for R2.0H2CL3 it was 0.002243 and for R2.0H2CL4 it was 0.0026).

### 3.3. Parametric Study

**Figure 16:**
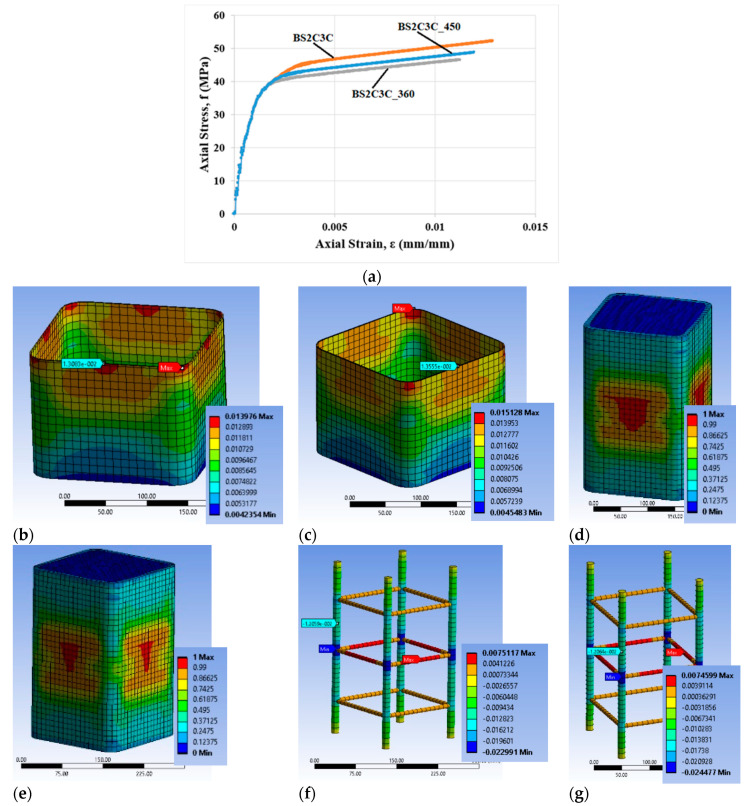
(**a**) FE analysis predictions versus experimental stress-strain results for the parametric study of specimen BS2CL3; variation of the CFRP strain and characteristic values of specimen (**b**) BS2C3C_360 at the level of the middle stirrup and (**c**) BS2C3C_450 at the level of the middle stirrup; variation of concrete damage of specimen (**d**) BS2C3C_360 at the ultimate condition and (**e**) BS2C3C_450 at the ultimate condition; variation of ε_stirrup_max_, ε_long_mid_ and ε_long_min_ for specimen (**f**) BS2C3C_360 and (**g**) BS2C3C_450.

The presented FE analyses did not deal with the effect of the quality of steel reinforcement on the effectiveness of the external FRP confinement. Additional FE analyses were performed for conventional longitudinal bars of 14 mm diameter with nominal yield strengths: 360 MPa, 450 MPa and 560 MPa. Figure 16a presents the FE analytical results for the BS2C3C specimen with the 3 layers of CFRP. The other features of the specimen remained the same. The nomenclature of the analyses was as follows:

- BS2C3C (the original specimen with yield stress of longitudinal steel 560 MPa).
- BS2C3C_360 (yield stress of longitudinal steel 360 MPa).
- BS2C3C_450 (yield stress of longitudinal steel 450 MPa).

All different analyses were terminated for the same ultimate tensile strain of the FRP at the corner, at the level between two stirrups, as assessed for specimen BS2C3C (0.009179). For specimen BS2C3C_360, the ultimate stress from the FE analysis was 46.5 MPa at a strain of 0.01125 and specimen BS2C3C_450 provided analytical stress of 48.71 MPa at a strain of 0.01195.

The analytical elaboration for the specimens BS2C3C_360 and BS2C3C_450 show that the material that finally fails was again the concrete, as in specimen BS2C3C. For BS2C3C_360, the tensile FRP strain at the ultimate condition, at the corner region of the mid-height stirrup was 0.01308 and at the corner of this stirrup it was 0.01398 (Figure 16b). For specimen BS2C3C_450, the FRP strain at the corner region of the mid-height stirrup was 0.01355 and at the corner of this stirrup was 0.01513 (Figure 16c). Figure 16d,e shows the critical area where the highest degree of damage occurred. At this final step, the strain of the FRP was close to the material failure in the FE analyses, but it did not exceed it.

It is also observed that the differences in the characteristic values for the strain of the FRP and longitudinal and transverse steel reinforcements among these specimens were marginal. Figure 16f,g highlights the corresponding deformational state of the steel cage for this series. It seems that the maximum compressive strain on the slender bars was far higher (around 2 times) than the concrete average axial strain at the ultimate condition and located at the stirrup level. In particular, for specimen BS2C3C_360 this strain was −0.02299 and for BS2C3C_450 it was −0.02447. Thus, the axial strains of the bars at the level in-between the stirrups were close to the average axial strains of concrete. In particular, for specimen BS2C3C_360 the strain was −0.012059 and for BS2C3C_450 it was −0.01206. The stirrup tensile strains were far beyond the yielding in all cases. For specimen BS2C3C_360 the strain was 0.007512 and for BS2C3C_450 it was 0.00746.

Comparing the FE results of these three RC columns, it seems that the axial stress-strain capacity of the columns was marginally decreased due to the lower yield stress of the steel bars. The bars of lower yield stress marginally affected the axial deformability levels of the columns, exhibiting a decrease of axial strain ductility.

### 3.4. FE Models for Columns Confined with Composite Ropes

**Figure 17:**
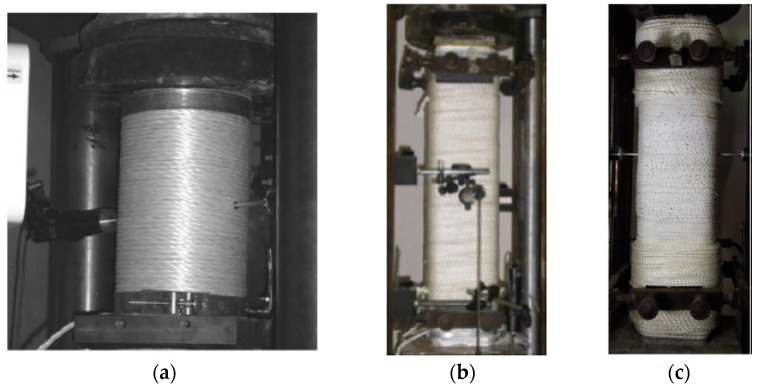
(**a**) Specimen VinL1v1R1 by [12] after the final cycle of loading; (**b**) specimen 500PPL4 by [13] before loading and (**c**) specimen RCPPL4 by [47] before loading.

Analyses were performed on cylindrical columns confined with Ropes of Polypropylene by Rousakis [12] (Figure 17a). These concrete cylinders had a 150 mm diameter and 300 mm height. The average cylindrical concrete strength during the tests was 15.56 MPa.

The vinylon fiber ropes (VFRs) of the study consisted of three Z-twisted fiber strands (Kurasoku Kensetsu Consultant Co. Ltd., Tokyo, Japan). They had a low E-modulus of 15,900 MPa, a tensile strength of f_VFR_ = 738 MPa and a tensile elongation at a failure of 4.6%. The first column had one layer with 5.83 mm spiral spacing (v1, full confinement), the second one had one layer with 13.18 mm spiral spacing (v2, one rope section, clear spiral spacing) and the third had one layer with 20.06 mm spiral spacing (v3, two rope sections, clear spiral spacing). The columns of the v1 group had stopped early for safety reasons before the fracture of the confining means.

The concrete was modeled using the RHT concrete model. The compressive strength for specimen VinL1v1R1 was 17.9 MPa and the tensile strength ratio f_t_/f_c_ was 0.1 (where f_t_ is the tensile strength and f_c_ is the compressive strength [75]). The composite rope was modeled as an equivalent hexahedral solid element with a “frictionless” interaction with the concrete core. For the properties of the ropes, an isotropic elasticity model was used. The modulus of elasticity of vinylon was 15,900 MPa in all the directions and the Poisson’s ratio was 0.3.

**Figure 18:**
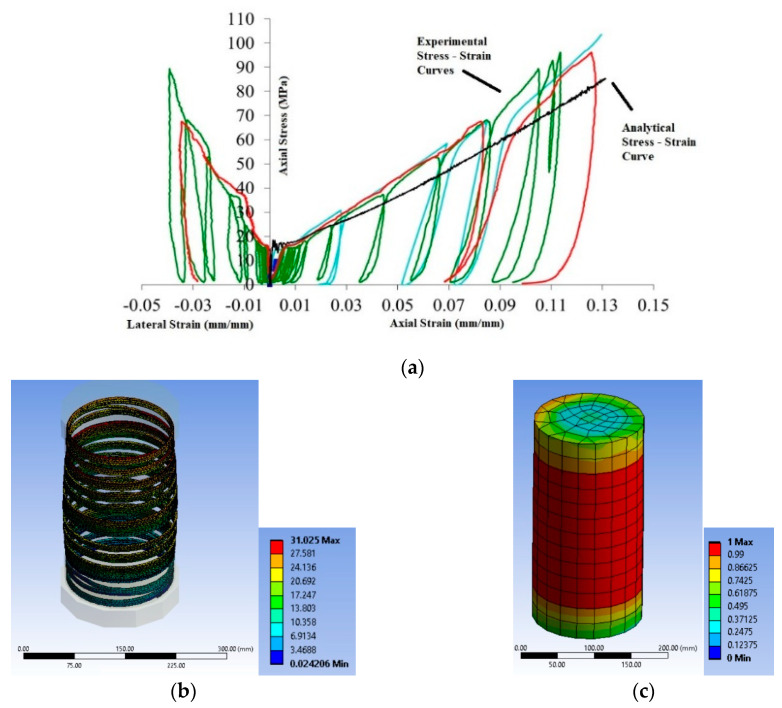
(**a**) Experimental vs. analytical stress-strain curves for columns of the VinL1v1 group wrapped with one full layer of Vinylon Fiber Ropes (VFR); (**b**) deformation of composite ropes for column VinL1v1 and (**c**) variation of damage of the concrete core for columns VinL1v1.

The analytical stress-strain curve for specimens of the VinL1v1 group subjected to axial compression is depicted in Figure 18a. The FE analysis may capture satisfactorily the general stress-strain behavior of the column, the temporary load drop and stabilization after extensive concrete cracking and the subsequent load increased up to very high axial strains. Figure 18b shows the total deformation of the composite rope at the stage of rope fracture and Figure 18c shows the damage variation of the concrete core after failure.

Finally, analyses were carried out on square RC columns confined with ropes of polypropylene by [13,47] (Figure 17b,c). These specimens had a 150 mm side and 750 mm height. They had internal longitudinal steel bars 4Φ8 (B500C) and transverse Φ5.5/100 mm (S220). They were externally wrapped with 4 layers of elastic polypropylene fiber rope (PPFR, products of Thrace Plastic Co. S.A, Alimos, Greece) of ultra-high deformability. For specimen 500PPL4, the average cylindrical concrete strength during the tests was 14.2 MPa and had sharp corners. Specimen RCPPL4 had the average cylindrical concrete strength, during the tests of the column, and was 19 MPa and the corner radius of the section was 17 mm.

For the simulation of PPFR, which is a Z-twisted two-strand rope, the ultimate stress was 405.3 MPa, the tensile modulus of elasticity was 2.0 GPa and 20.36% elongation at failure. The yield strength of the longitudinal steel and stirrups was f_y,long_ = 500 MPa and f_y,stirrup_ = 220 MPa, respectively. The modulus elasticity of steel was Es = 200,000 MPa and the Poisson’s ratio ν = 0.3.

**Figure 19:**
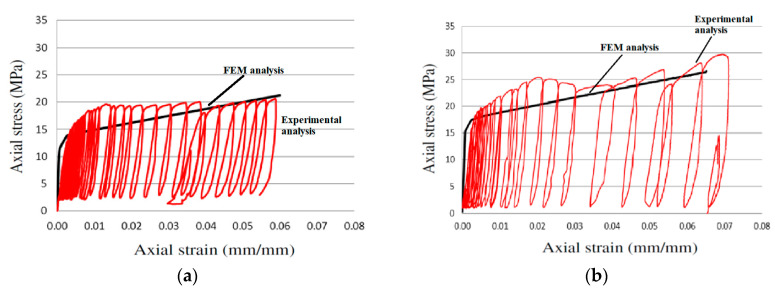
Experimental vs. analytical stress-strain curves for column (**a**) 500PPL4 by [13] wrapped with four full layers of polypropylene FR and (**b**) RCPPL4 by [47] wrapped with four full layers of polypropylene FR.

Figure 19a shows the analytical stress-strain curve for specimen 500PPL4 subjected to axial compression and Figure 19b shows the analytical stress-strain curve for specimen RCPPL4 subjected to axial compression.

## 4. Conclusions

Three-dimensional finite-element modeling was used herein to study the axial mechanical behavior of square and rectangular RC columns, confined with FRP jackets and continuous composite ropes. The pseudodynamic approach was utilized for the first time to enable for the reproduction of monotonic, repeated or cyclic imposed displacements (usually met in seismic resistant assessment or retrofit of columns). It may capture demanding inelastic behaviors, damage redistribution effects and local degrading behavior. The analytical models of the small and real scale columns under investigation were developed based on suitably calibrated material models, realistic representation of all the internal steel detailing and the external FRP jacketing, suitably designed FE meshing and interactions among different materials and interfaces and proper body interactions (necessary for the pseudodynamic process). The analytical results of RC columns confined with composites compared well with the experimental stress-strain curves of several characteristic RC columns of the square or rectangular concrete section with varying concrete strength, corner radius, FRP jacket mechanical properties or non-bonded composite rope confinement. In particular, the FE analyses follow closely the experimental curves for specimen series BS1C/BS2C and LSR-R-1-3 with ascending curves and for specimen series R2.0H2CL with descending second branches. All the analyses were stopped at the experimental ultimate axial displacement while the corresponding ultimate axial stress in all cases exhibited an AAE of 13%. Therefore, the proposed validated pseudodynamic modeling approach enabled for the first time to investigate analytically several critical design parameters and conduct parametric analyses.

The advanced FE models considered the effects of the slenderness of bars, deficient stirrups or local crushing of the concrete and therefore were used as a basis to investigate the effects of steel reinforcement quality on the stress-strain behavior of the columns. Several parametric analyses were investigating the influence of different yield stress of longitudinal steel and stirrups (360 MPa, 450 MPa and 560 MPa). It is observed that the bars of lower yield stress affected marginally the axial deformability levels of the columns, exhibiting a decrease of axial strain ductility.

FRP jacket fracture is widely accepted as the failure criterion for most of the experimentally investigated cases and for the proposed design models. Further, the contribution of steel bars and stirrups may be variable. This study focused on the significant variation of the field of developed strains on the FRP jacket at the ultimate condition and on the developed strains and deformations on steel cages among different columns. Eventually, all steel bars under compression exhibited and maintained strains higher than the yield one with high variation along the axis. All stirrups under tension exceeded their yield strain or achieved strains very close to it. The measured deformations suggest nonuniform bulging of the retrofitted column both axially and transversely. The critical position to place strain gauges in order to measure the representative axial strain of the steel reinforcement under axial compression in experiments (close to the average one for the column), may be in the middle-height of the area of interest (far from end boundaries) and at the mid-distance between successive stirrups (probably two or four strain gauges, symmetrically placed, are the best option if the bar buckles significantly) and not at the area close to the stirrups. The investigated variable field of FRP strains suggest that there was, in some cases, local fracture of the jacket at the corner region (even local concrete crushing at the corner region) while the global behavior of the column did not reach the ultimate failure. This aspect was captured due to the suitable composite jacket modeling among others. The measured FRP tensile strains were higher at the corner region at the height of the stirrups for most of the specimens. For lower FRP confinement and for lower corner radius there was a high local damage development in the concrete and local bulging in both the concrete and FRP. It seems there was an interaction between the local damage of the concrete and the FRP fracture that needs further investigation. Finally, the advanced parametric FE analyses confirm previous investigations [66,67,68] and better quantify the marginal but earlier failure of the retrofitted RC columns when the yield stress of the longitudinal bars was lower.

All these investigated critical parameters will significantly enrich the extensive experimental databases for such retrofitted columns gathered in [70,71]. The analytical values retrieved from this study are believed to address missing critical parameters in an advanced experimental-analytical database (hybrid approach, see [74]) for retrofitted RC columns under axial compression and provide more reliable models for failure axial strains.

## Figures and Tables

**Table 1:** Geometrical and nominal mechanical data for the analyzed columns.

Specimen	h (mm)	b (mm)	H (mm)	r_c_ (mm)	Long.	f_y,long_ (MPa)	Stir.	f_y,stir_ (MPa)	f_co_ (MPa)	*n*	t_FRP_	E_FRP_ (GPa)
**BS1C1C [2]**	200	200	320	30	4Φ14	500	Φ8/200	500	25.5	1	0.117	240
**BS1C3C [2]**	200	200	320	30	4Φ14	500	Φ8/200	500	25.5	3	0.117	240
**BS1C5C [2]**	200	200	320	30	4Φ14	500	Φ8/200	500	25.5	5	0.117	240
**BS2C1C [2]**	200	200	320	30	4Φ14	500	Φ8/95	500	25.5	1	0.117	240
**BS2C3C [2]**	200	200	320	30	4Φ14	500	Φ8/95	500	25.5	3	0.117	240
**BS2C5C [2]**	200	200	320	30	4Φ14	500	Φ8/95	500	25.5	5	0.117	240
**LSR-R-1-3-10b [20]**	250	250	500	10	4Φ14	345	Φ8/200	476	10.83	3	0.165	230
**LSR-R-1-3-20b [20]**	250	250	500	20	4Φ14	345	Φ8/200	476	10.83	3	0.165	230
**LSR-R-1-3-40b [20]**	250	250	500	40	4Φ14	345	Φ8/200	476	10.83	3	0.165	230
**R2.0H2CL3 [28]**	400	200	1000	40	8Φ16	360	Φ8/100	345	46.3	3	0.167	240
**R2.0H2CL4 [28]**	400	200	1000	40	8Φ16	360	Φ8/100	345	46.3	4	0.167	240

**Table 2:** Mechanical data used in the 3-dimensional finite element (FE) models.

Specimen	f_co,FE_ (MPa)	f_y,long,FE_ (MPa)	f_y,long,FE_ (MPa)	E_FRP,gt,FE_^1^ (MPa)	E_FRP,ga,FE_^2^ (MPa)*	t_FRP,FE_	Poisson Ratio FE	Shear Modulus FE (MPa)	ε_FRP,FE_
**BS1C1C [2]**	25.5	600	600	59,160	7500	0.475	0.3	17,500	0.015
**BS1C3C [2]**	25.5	600	600	59,160	7500	1.424	0.3	17,500	0.015
**BS1C5C [2]**	25.5	600	600	59,160	7500	2.373	0.3	17,500	0.015
**BS2C1C [2]**	25.5	600	600	59,160	7500	0.475	0.3	17,500	0.015
**BS2C3C [2]**	25.5	600	600	59,160	7500	1.424	0.3	17,500	0.015
**BS2C5C [2]**	25.5	600	600	59,160	7500	2.373	0.3	17,500	0.015
**LSR-R-1-3-10b [20]**	10.83	345	476	59,160	7500	1.924	0.3	17,500	0.015
**LSR-R-1-3-20b [20]**	10.83	345	476	59,160	7500	1.924	0.3	17,500	0.015
**LSR-R-1-3-40b [20]**	10.83	345	476	59,160	7500	1.924	0.3	17,500	0.015
**R2.0H2CL3 [28]**	46.3	360	345	59,160	7500	2.032	0.3	17,500	0.015
**R2.0H2CL4 [28]**	46.3	360	345	59,160	7500	2.710	0.3	17,500	0.015

^1^ E_FRP,gt,FE_ is the elastic modulus in the global transverse axis; ^2^ E_FRP,ga,FE_ is the elastic modulus in the global axial axis.
